# The Decadence of the "Civilised" Negro

**Published:** 1902-09-20

**Authors:** 


### THE DECADENCE OF THE "CIVILISED" NEGRO.

Dr. Perry,[1] pathologist to the State Sanatorium, Milledgeville, has recently given some striking figures showing how great has been the increase in insanity among the negroes in certain Southern States of North America since the abolition of slavery. According to the census of 1860 there were in the State of Georgia 44 insane negroes, the negro population being at that time 465,698. The ratio of insane to the total population was thus 1 to 10,584. In 1870 the ratio was 1 to 4,225; in 1880 it was 1 to 1,764; in 1890 it was 1 to 1,533; while in 1900 it was estimated to be 1 in 1,149. The census of 1900 gives the negro population of Georgia as 1,034,813. Thus in forty years the total negro population in the State has been a little more than doubled, while the number of insane has increased twenty-fold. No such rapid and radical change in the mental stability of a race is recorded in history, and this outburst of insanity becomes still more remarkable when we consider that for generations prior to 1860 the coloured people had been free from mental disease. It has developed, we are told, without the slightest hereditary taint. But this mental decadence, which has fallen upon the negro since he was left to his own devices, by no means stands alone. There seems to be good evidence that in "the old days," whatever else might be said about him, the negro was a splendid specimen physically. But we are told that this is not the case now, and that, in fact, freedom has brought about a great physical deterioration of the race. Among other indications is to be noted the alarming prevalence of tuberculosis among the negro population of to-day, a prevalence which is doubtless the result of their rush to the large towns and their readiness to live in overcrowded houses in conditions provocative of that disease. None the less does it show the tendency of the negroes to decadence, and one mode of it, for during the days of slavery "the disease was so seldom found among them that they were considered almost immune to it. In fact, some of the older writers took that stand squarely, and asserted that consumption was unknown in the race." From being thus so rare as to be almost unknown, it has in a single generation become so prevalent that more negroes in the South are dying of tuberculosis than of any other disease. But the causes of the decadence are manifold. Accepting a lower standard, with strong natural bent towards indulgence of the appetites and a large endowment of animal passions, while judgment and a consideration of consequences are usually deficient in them, the negroes have become lax in their morals, with the natural result of a widespread prevalence of venereal disease among them, and have been a ready prey to the deteriorating influences of town life, including, of late years, a large resort to drugs, such as opium and cocaine. How far the reports we receive from time to time from the Southern States are to be regarded as strictly impartial we are unable to say, but springing as they do from many sources, we cannot but feel that they deserve special consideration by English people at the present day. In the settlement of South Africa it will not be long before we shall have to face this most embarrassing negro question, and have to decide whether we will, by keeping the negro in subjection, throw overboard a not inconsiderable store of catch-words of which as a nation we are very proud—among which will be that concerning the freedom which belongs to everyone who treads on British soil, and all the rest of it—or by giving the said freedom to the negro, by acknowledging him as a man and a brother, and by allowing him to "take his place in the world," ensure his mental and physical decadence, even though in mere numbers he may multiply exceedingly under the protection of our laws.

[1] New York Med. Rec.

